# A gene-based predictive model for lymph node metastasis in cervical cancer: superior performance over imaging techniques

**DOI:** 10.1186/s12967-025-06327-3

**Published:** 2025-04-03

**Authors:** Dongdong Xu, Xibo Zhao, Dongdong Ye, Chuying Huo, Xuanwei Peng, Yunyun Liu, Huaiwu Lu

**Affiliations:** 1 Department of Gynecological Oncology, Sun Yat-Sen Memorial Hospital, Sun Yat-Sen University, Guangzhou, Guangdong China; 2 Guangdong Provincial Clinical Research Center for Obstetrical and Gynecological Diseases, Guangzhou, Guangdong China

**Keywords:** Lymph node metastasis, Cervical cancer, Predictive model, Gene expression

## Abstract

**Objective:**

Lymph node metastasis (LNM) critically impacts the prognosis and treatment decisions of cervical cancer patients. The accuracy and sensitivity of current imaging techniques, such as CT and MRI, are limited in assessing lymph node status. This study aims to develop a more accurate and efficient method for predicting LNM.

**Methods:**

Three independent cohorts were merged and divided into training and internal validation groups, with our cohort and those from other centers serving as external validation. A predictive model for LNM in cervical cancer was established using the LASSO regression and multivariate logistic regression. The diagnostic performance of the predictive model was compared with that of CT/MRI in terms of accuracy, sensitivity, specificity, and AUC.

**Results:**

Using RNA-seq data, four independent predictive genes (MAPT, EPB41L1, ACSL5, and PRPF4B) were identified through LASSO regression and multivariate logistic regression, and a predictive model was constructed to calculate the LNM risk score. Compared with CT/MRI, the model demonstrated higher diagnostic efficiency, with an accuracy of 0.840 and sensitivity of 0.804, compared to CT/MRI’s accuracy of 0.713 and sensitivity of 0.587. The predictive model corrected 81% of misdiagnoses by CT/MRI, demonstrating significant improvements in accuracy and sensitivity.

**Conclusion:**

The predictive model developed in this study, based on gene expression data, significantly improves the preoperative assessment accuracy of LNM in cervical cancer. Compared to traditional imaging techniques, this model shows superior sensitivity and accuracy. This study provides a robust foundation for developing precise diagnostic tools, paving the way for future clinical applications in individualized treatment planning.

**Supplementary Information:**

The online version contains supplementary material available at 10.1186/s12967-025-06327-3.

## Introduction

Cervical cancer ranks as the second most prevalent malignancy in women globally, coming after breast cancer. The World Health Organization (WHO) reports that each year, around 600,000 new cases are diagnosed, and approximately 300,000 fatalities occur, predominantly affecting populations in developing nations [1]. The incidence and progression of cervical cancer are strongly linked human papillomavirus (HPV) infection [2]. Although screening and vaccination have advanced significantly, the incidence of cervical cancer continues to rise annually in China, with younger women increasingly affected, severely impacting the survival and quality of life of women of childbearing age [3].

For individuals diagnosed with early-stage cervical cancer, surgery often leads to a favorable prognosis, with a 5-year survival probability between 85 and 90%. However, once LNM occurs, the cancer progresses to stage III, and the prognosis worsens significantly, with the 5-year survival probability dropping to 50–55% [4, 5]. LNM is also a crucial factor in determining treatment strategies. The National Comprehensive Cancer Network (NCCN) guidelines advocate surgery as a treatment option for individuals without lymph node metastasis (LNM), while for those with metastasis, chemoradiotherapy is preferred [6]. Inaccurate preoperative lymph node assessment can lead to multiple treatments and increased treatment-related complications. Moreover, lymph node status affects decisions regarding fertility preservation and ovarian preservation in early-stage cervical cancer. If LNM is present, fertility and endocrine functions cannot be preserved. The NCCN guidelines clearly state that for patients undergoing fertility-preserving surgery, if lymph node positivity is detected intraoperatively, the surgery should be aborted, and radical treatment, such as chemoradiotherapy, should be initiated. Continuing fertility-preserving surgery under such circumstances may lead to inadequate treatment [6].

Currently, imaging techniques like computed tomography (CT), magnetic resonance imaging (MRI), and positron emission tomography (PET) are commonly utilized to assess LNM [7]. A comprehensive review indicated that CT scans have a 43% sensitivity in detecting LNM, whereas MRI scans demonstrate a 60% sensitivity. Consequently, the relatively low sensitivity of both imaging methods in assessing LNM in cervical cancer may result in overlooked diagnoses. In contrast, PET scans have a sensitivity of 74.7% and specificity of 97.6%. However, the high cost and associated challenges of PET scanning limit its widespread use, especially in low-income countries [8, 9]. Intraoperative assessments, such as sentinel lymph node biopsy, have a diagnostic accuracy of 77.4% for detecting LNM [10]. However, it is worth noting that sentinel lymph node biopsy requires general anesthesia, is invasive, and is costly [11].

Therefore, finding a more accurate, efficient, and cost-effective method for predicting LNM in cervical cancer has become a research hotspot. While some studies have attempted to use serum markers and molecular biological markers for prediction, these methods have not yet been widely validated and applied. In response to the limitations of current methods, gene-based prediction models provide unique advantages in precision medicine, offering higher accuracy and the potential for individualized treatment strategies. By analyzing genetic expression profiles derived from a substantial group of cervical cancer patients, this study aims to develop a clinically practical predictive model and retrospectively validate it, providing a new tool for preoperative LNM assessment. The establishment of this model not only helps to improve preoperative diagnostic accuracy but also provides a scientific basis for the formulation of individualized treatment plans, with the potential to enhance the prognostic outcomes for individuals with cervical cancer.

## Methods

### Data collection and processing

This study's datasets include cervical cancer RNA-seq data and clinical data. RNA sequencing data were sourced from The Cancer Genome Atlas (TCGA) database (TCGA_CESC cohort) via Xena Browser and from The Gene Expression Omnibus (GEO) database, specifically for the GSE7410 and GSE26511 cohorts. Patients with clinical data that did not match the sequencing data and those without available lymph node status were excluded. In total, 272 patients were ultimately considered for the final analysis. Additionally, postoperative pathological tissue samples were collected from 94 patients at Sun Yat-sen Memorial Hospital (SYSMH) and 57 patients at a tertiary hospital to serve as external validation cohorts. Lymph node status is confirmed after undergoing radical hysterectomy, pelvic lymphadenectomy, and para-aortic lymphadenectomy. Positive lymph nodes are classified as N1 or N2, while negative lymph nodes are classified as N0.This research followed the established ethical standards set forth in the 2008 Declaration of Helsinki and received approval from the Ethics Committee of SYSMH (SYSKY-2024-707-01). Written informed consent was obtained from all participants before their involvement in the study.

**Fig. 1:**
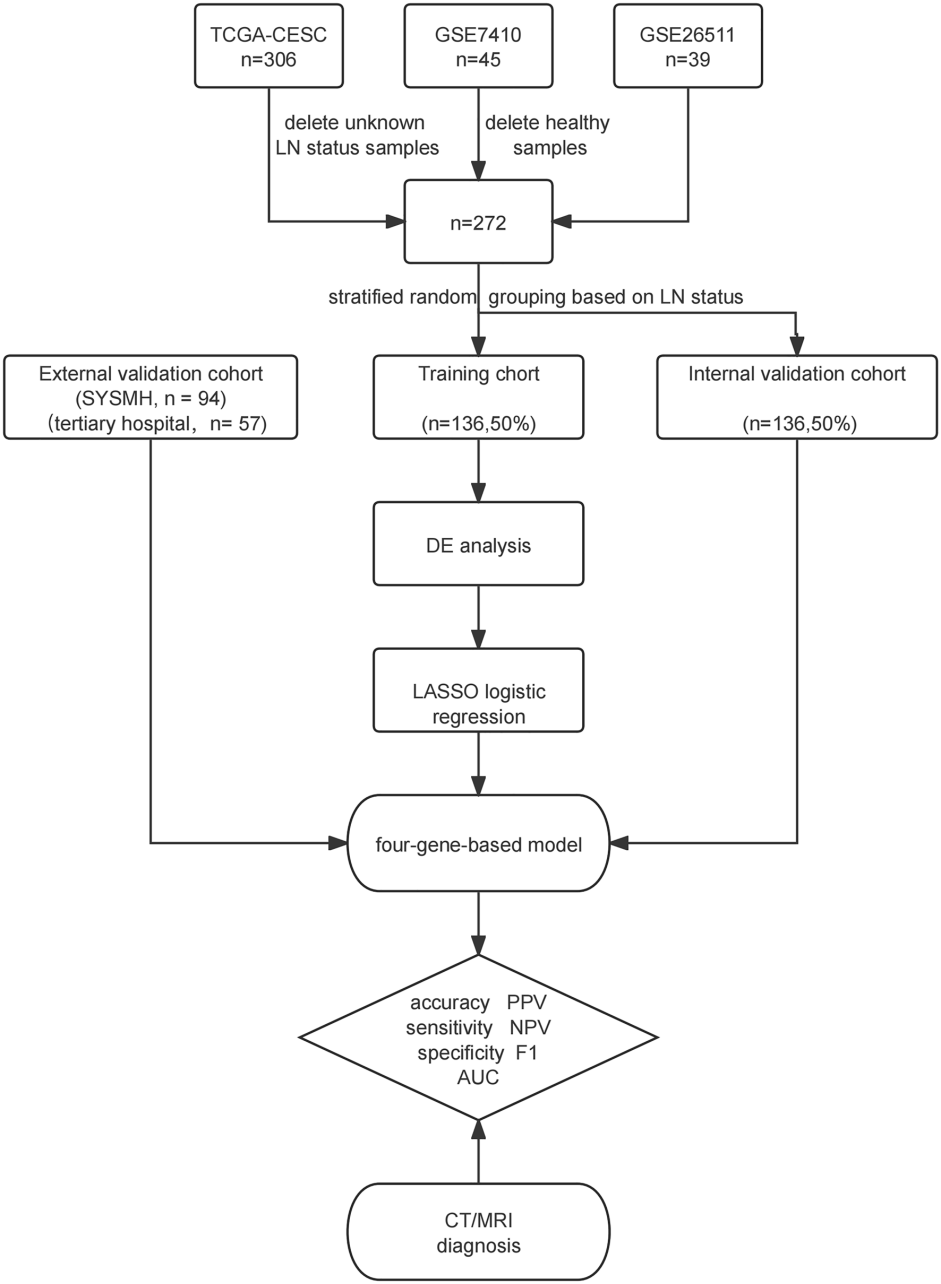
Flow chart in this study. LN: lymph node; LASSO: least absolute shrinkage and selection operator; PPV: Positive Predictive Value; NPV: Negative Predictive Value; F1: F1 score; AUC: area under roc curve

RNA-seq data were processed as follows: Common genes were retained to integrate the three cohorts, and low-abundance genes not detected in more than one-fourth of the samples were removed. Gene expression profiles were converted into Transcripts Per Million (TPM) and subsequently analyzed using log2(TPM + 1) transformation. To eliminate batch effects caused by non-biological technological variations, we applied the ‘Combat’ algorithm using the R package ‘sva,’ ensuring consistent gene expression levels across different datasets [12]. Adjusted expression profiles were validated through Principal Component Analysis (PCA) (Supplementary Fig. 1A-B). Ultimately, the combined expression profiles were randomly allocated into training and internal validation groups (Fig. 1).

### Differential expression analysis

The R package “limma” was applied for differential expression analysis [13]. To maximize the identification of potential LNM markers, differentially expressed genes (DEGs) were characterized as those with a P-value below 0.05 and a log2(fold change) greater than 0.5.

### Model construction

Variable selection and complexity regularization were conducted using the least absolute shrinkage and selection operator (LASSO) regression to prevent overfitting. LASSO analysis was carried out with the R package ‘glmnet’ [14]. Key genes were selected from LASSO regression outcomes, with genes showing p < 0.05 in multivariate logistic regression retained for model construction. To generate the predictive model, multivariate logistic regression was performed again using the selected independent predictive genes. A nomogram was developed as a visual tool for calculating RNA-seq-based risk scores. The individual risk score for each patient was determined using the formula: ${\text{Risk score}} = \alpha  {\sum \nolimits ^{{{N}}} _{i=1}}\left( {{\text{Exp}}({\text{i}}) \times {\text{Coef}}({\text{i}})} \right) + \beta$, where “Exp” denotes the expression level of gene i in the model, and “Coef” represents its corresponding regression coefficient. The parameters α and β are specific constants associated with the nomogram.

### Model evaluation

The performance of the predictive model was assessed based on calibration, discrimination, and practical utility. Calibration curves depicted the agreement between observed outcomes and predictions using the R package “rms”. The predictive model's discriminatory performance was assessed using ROC curves, generated with the R packages ‘pROC’ and ‘ggplot2’ [15]. Its clinical utility was further evaluated via decision curve analysis (DCA) utilizing the ‘rmda’ package. DCA helps determine whether integrating the predictive model into clinical decision-making provides a benefit to patients.

### Real-time quantitative polymerase chain reaction (RT-qPCR)

Total RNA was isolated from cancer tissues using TRIzol (Invitrogen), and subsequently, reverse transcription was carried out with the PrimerScript RT-PCR kit. qPCR was conducted with the SYBR Green reaction mix on the LightCycler 480 system. The 2 − ΔCt method was employed to determine relative expression levels, using β-actin as the internal reference. A full list of primers is available in Supplementary Table S1.

### Diagnostic efficiency

All patients at SYSMH underwent preoperative CT or MRI examinations. CT/MRI imaging parameters included lymph node size and morphology, enhancement, and necrotic characteristics. Skilled radiologists analyzed the images and made clinical diagnoses of LNM. Pathologists confirmed pathological LNM. The performance of the model and CT/MRI in diagnosis was assessed through the Receiver Operating Characteristic-Area Under the Curve (ROC-AUC), along with sensitivity, specificity, PPV, NPV, accuracy, and F1 scores.

### Independent prognostic analysis

To evaluate the predictive model's clinical prognostic significance, we gathered clinical data from the TCGA-CESC cohort (including age, Body Mass Index (BMI), Tumor (T), Node (N), stage, grade, and histological type) and grouped the data accordingly. We also collected sample data from SYSMH, grouping samples by N stage, myometrial invasion, and lymphovascular invasion status (LVSI), and further subdividing deep myometrial invasion and lymphovascular invasion-positive samples based on lymph node status. Analysis of variance (ANOVA) and t-tests were used to compare risk scores between groups, and box plots were created utilizing the R package “ggpubr”. Additionally, we performed ROC analysis on risk scores for deep myometrial invasion and lymphovascular invasion-positive samples from SYSMH.

To determine if the model functions as an independent prognostic indicator, clinical information from SYSMH (including age, BMI, CT/MR results, myometrial invasion, histological type, grade, lymphovascular invasion, T, M, N, and stage) were integrated with risk scores for both univariate and multivariate logistic regression analyses.

### Gene ontology (GO) and Kyoto encyclopedia of genes and genomes (KEGG) enrichment analyses

The R package ‘limma’ was utilized to conduct DE analysis of mRNA data between two risk groups (|logFC|> 0.5, padj < 0.05), identifying DEGs specific to each group. Functional and pathway enrichment analyses were then performed using the Metascape database (https://metascape.org/gp/index.html).

### Statistical analysis

Demographic data were reported as mean ± standard deviation (SD) for normally distributed continuous variables, whereas non-normally distributed continuous variables were expressed as mean with interquartile range (IQR). Categorical variables were summarized as frequency and percentage. The Mann–Whitney test was applied for comparisons between two groups with unequal variances. Kaplan–Meier (K–M) survival curves, along with log-rank tests, were utilized to evaluate the prognostic significance of the predictive model. All statistical analyses were conducted using R software (version 4.3.2, https://www.r-project.org/). All comparisons were two-tailed, and a significance level of 0.05 was used unless otherwise specified.

## Results

### Characteristics of patients from public databases

Figure 1 presents the flowchart of this study. We included 193 patients from the TCGA_CESC cohort, 40 patients from the GSE7410 cohort, and 39 patients from the GSE26511 cohort. Among the 272 cervical cancer patients, 98 (36.03%) were diagnosed with LNM. After random grouping, model construction was based on data from 136 patients in the training cohort.

### Identification of genes associated with LNM in cervical cancer

**Fig. 2:**
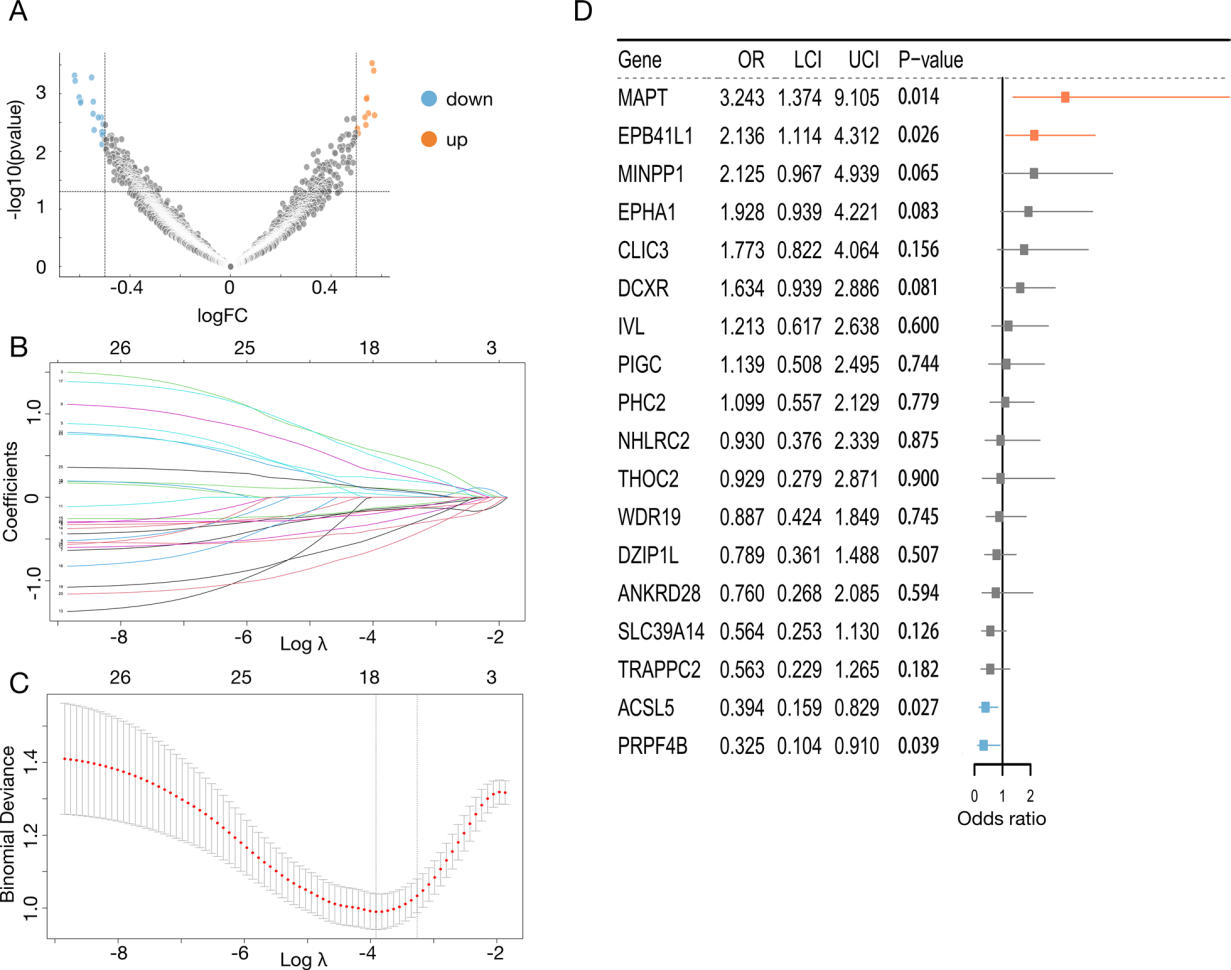
Screening key genes predicting lymph node metastasis. **A** DEGs between lymph node-positive patients and lymph node-negative patients. **B** The curve of the coefficient path of 32 lymph node metastasis-related genes identified by the LASSO regression in the training cohort. **C** The selection of the adjustment penalty parameter λ in the LASSO model via tenfold cross validation according to the error within one standard error range of the minimum. **D** Multivariate logistic regression for 18 genes to identify the key genes that can independently predict lymph node metastasis. OR: odd ratios; LCI: lower confidence interval; UCI: upper confidence interval

Initially, key DEGs were identified by comparing lymph node-positive and negative patients. Among those with LNM, 10 genes exhibited significant upregulation, whereas 16 genes were downregulated. These DEGs were considred as potential markers for LNM (Fig. 2A and Supplementary Table 2). Using LASSO regression with a penalty parameter of λ = 0.0199, 18 genes were selected to optimize model performance (Fig. 2B, C). Further analysis through multivariate logistic regression was conducted to identify independent predictive genes (Fig. 2D and Supplementary Table 3). Finally, four genes were included in the LNM predictive model. Among them, MAPT and EPB41L1 were risk genes for LNM, while ACSL5 and PRPF4B were protective genes.

### The four-gene model for predicting LNM exhibits good performance

**Fig. 3:**
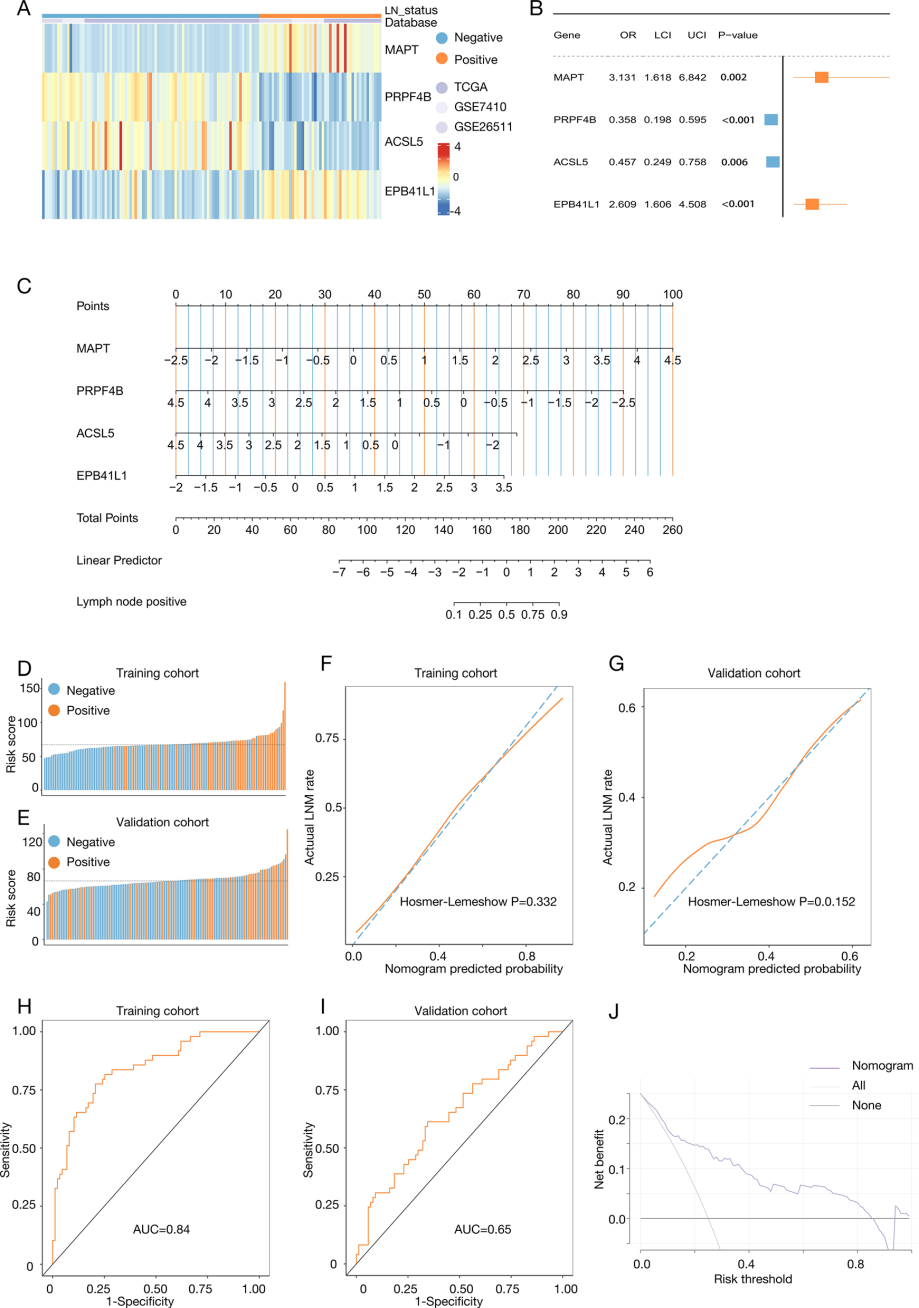
Establishing and evaluating the lymph node metastasis prediction model. **A** Expression profile of the four genes associated with lymph node status. **B** Four genes were employed to establish the prediction model according to multivariate logistic regression coefficients. **C** The nomogram assembled with MAPT, PRPF4B, ACSL5 and EPB41L1 to predict lymph node metastasis. **D**, **E** Density distribution plots of lymph node metastasis events in patients with individual risk scores in the d training and validation cohort. **F**, **G** The calibration curves of the prediction model for the training and validation cohort. **H**, **I** The ROC curves of the prediction model for the training and validation cohort. **J** Decision curve analysis of the prediction model. OR: odd ratios; LCI: lower confidence interval; UCI: upper confidence interval; AUC: area under roc curve

A heatmap showed the expression characteristics of the four genes in cervical cancer patients (Fig. 3A). Significant differences in expression were observed among patients with and without LNM. Logistic regression was employed to develop the predictive model, determining the contribution of each gene to the risk score through regression coefficients (Fig. 3B). The nomogram, a visual scoring tool, standardizes each variable into a score. By summing these individual scores, the total score indicates the probability of LNM. Using the regression coefficients, we constructed a nomogram to estimate LNM risk (Fig. 3C). The individual risk score for each patient was determined using the formula:

$$\begin{aligned} {\text{Risk score}} = & ({\text{Exp }}\left( {{\text{MAPT}}} \right) \times {1}.{1412834} - {\text{Exp }}\left( {{\text{PRPF4B}}} \right) \times {1}.0{281673} - {\text{Exp }}\left( {{\text{ACSL5}}} \right) \times 0.{783}0{517} \\ & + {\text{Exp }}\left( {{\text{EPB41L1}}} \right) \times 0.{9589773} - 0.{9}0{18145)} \times {12}.{51724} + {173}.0{316}. \\ \end{aligned}$$

The nomogram facilitates LNM risk assessment based on RNA-seq data. We additionally validated the predictive value of the model, observing that LNM probability increased with risk score in both training and validation cohorts (Fig. 3D, E). Calibration curves showed minimal deviation between actual and predicted outcomes, with Hosmer–Lemeshow test p-values of 0.332 (training cohort, Fig. 3F) and 0.152 (validation cohort, Fig. 3G). The predictive model demonstrated robust discriminatory ability, with AUC values of 0.84 (95% CI 0.77–0.90) in the training cohort and 0.65 (95% CI 0.55–0.76) in the validation cohort (Fig. 3H, I). Decision curve analysis (DCA) indicated clinical applicability, suggesting patient benefit when LNM probability was below 0.83 (Fig. 3J).

### Comparative analysis of diagnostic efficiency: predictive model vs. CT/MRI

**Fig. 4:**
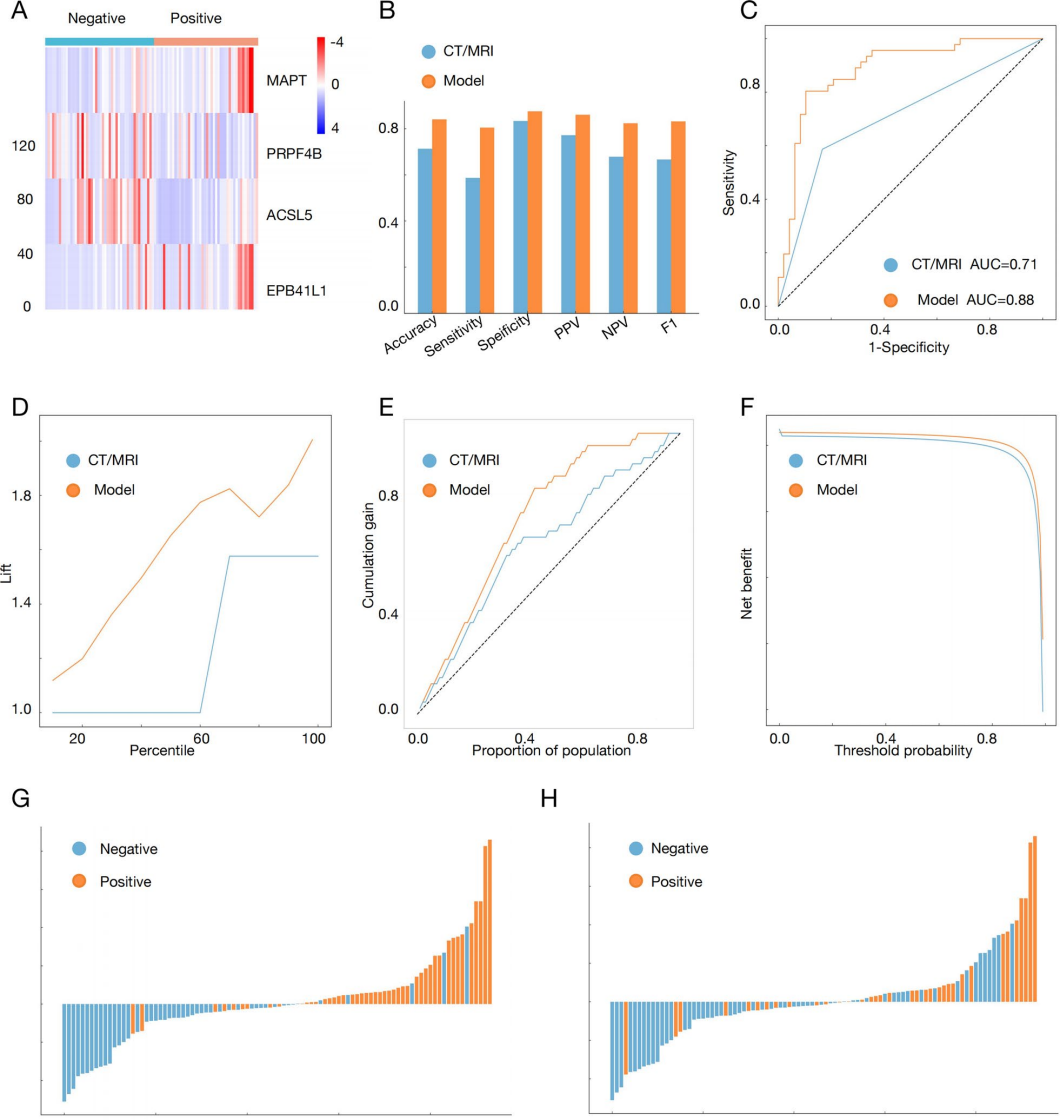
Comparison of the diagnostic efficacies of the Fast LN Scoring System and CT/MRI in predicting LN metastasis. **A** The gene expression heatmap of RT-qPCR validation of the external set. **B**–**F** The performance metrics, ROC curve, lift chart, cumulative gain chart and decision curve of CT/MRI imaging and prediction model. **G** Relationship between risk score and lymph node status. **H** Association of risk scores with CT/MR Diagnosis of lymph node status. PPV: Positive Predictive Value; NPV: Negative Predictive Value; F1: F1 score; AUC: area under roc curve. *p < 0.05, **p < 0.01, ***p < 0.001, ****p < 0.0001

To further validate the effectiveness of the model, we selected patients from our hospital. External validation samples represented diverse clinical backgrounds, including various tumor stages, histological subtypes, and LVSI, enhancing the model's generalizability. The clinical information can be found in Table S5. First, we used postoperative specimens to perform qPCR to obtain expression levels of the four key genes, which were consistent with the public database results (Fig. 4A). We then compared the diagnostic performance of the predictive model with CT/MRI. The performance metrics of the predictive model were as follows: accuracy of 0.840 (95% CI 0.8132–0.8690), sensitivity of 0.804 (95% CI 0.7637–0.8449), specificity of 0.875 (95% CI 0.8519–0.8933), positive predictive value (PPV) of 0.860 (95% CI 0.8355–0.8806), negative predictive value (NPV) of 0.824 (95% CI 0.8039–0.8440), and F1 score of 0.831 (95% CI 0.8104–0.8520). In comparison, the performance metrics of CT/MRI were as follows: accuracy of 0.713 (95% CI 0.7360–0.8031), sensitivity of 0.587 (95% CI 0.5149–0.6524), specificity of 0.833 (95% CI 0.8081–0.8594), PPV of 0.771 (95% CI 0.7432–0.8043), NPV of 0.678 (95% CI 0.6484–0.7101), and F1 score of 0.667 (95% CI 0.6360–0.7094). The data indicated that the predictive model outperformed CT/MRI in all metrics, particularly in sensitivity and accuracy, demonstrating better classification performance (Fig. 4B). Additionally, the AUC of the predictive model was 0.88 (95% CI 0.81–0.96), while the AUC of CT/MRI was only 0.71 (95% CI 0.62–0.80) (Fig. 4C). A higher AUC value indicates better performance in distinguishing positive and negative samples, suggesting higher diagnostic accuracy and stability of the model.

Lift charts and cumulative gain charts further validated this conclusion. In the lift chart, a higher curve indicates better identification of positive cases at specific percentiles. The cumulative gain chart visually shows cumulative gains at different population proportions, with a higher curve indicating better identification of positive cases in a specific population proportion. Decision curve analysis considers the cost of false positive and false negative, evaluating the model's effectiveness through net benefit, with a higher curve indicating greater net benefit at a given threshold. These three charts indicated that the predictive model outperformed imaging (CT/MRI) in predicting high-risk and positive cases, as well as in overall effectiveness (Fig. 4D–F).

Further analysis revealed that the predictive model corrected 81% of the misdiagnoses made by CT/MRI (22/27 patients), including all 8 pN0 patients (8/8) and 14 out of 19 pN1-2 patients (14/19) (Fig. 4G, H). Specifically, among the 19 false-negative samples identified by CT/MRI, the predictive model correctly classified 14 as positive. Furthermore, all 8 samples that were misdiagnosed as positive by CT/MRI were accurately classified as negative by the predictive model. Additionally, the risk score generated by the predictive model served as an independent factor in predicting LNM, whereas CT/MRI was not (Supplementary Table 4). This suggests that the predictive model could serve as an alternative diagnostic approach to CT/MRI.

To further validate the robustness of our model, we additionally included fifty-seven cervical cancer samples from another independent gynecology center for qPCR validation. The levels of expression for the four key genes were consistent with our previous findings (Supplementary Fig. 1C), and the AUC value reached 0.95 (95% CI 0.89–0.99) (Supplementary Fig. 1D), which further supports the reliability and stability of the model.

In summary, the predictive model significantly outperformed CT/MRI in diagnostic efficiency, particularly in sensitivity and accuracy, indicating higher potential and practical value in clinical applications.

### Validation and subgroup analysis of the LNM predictive model

**Fig. 5:**
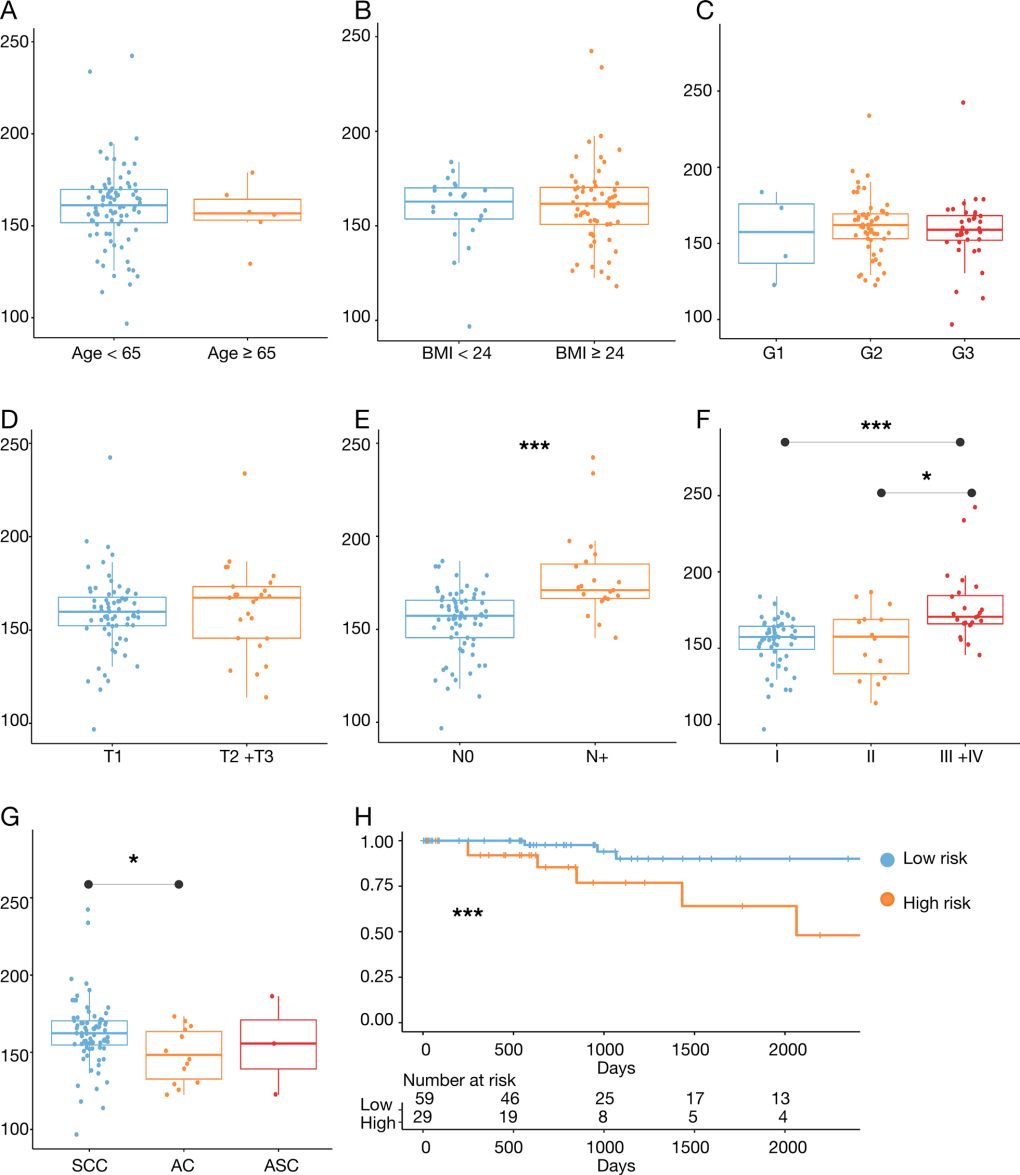
Subgroup analysis in the TCGA cohort. **A** Riskscore corresponding to each Age group (< 65 years old and ≧ 65 years old). **B** Riskscore corresponding to each BMI (< 24 and ≧ 24) group. **C** Riskscore corresponding to each differentiation degree (G1, G2 and G3). **D** Riskscore corresponding to each group of T stage (T1 and T2 + T3) **E** Riskscore corresponding to each group of N stage (N0 and N +). **F** Riskscore corresponding to each group of FIGO Stage (Stage I + II and Stage III + IV). **G** Riskscore corresponding to each group of pathological type (squamous carcinoma, adenocarcinoma and adenosquamous carcinoma). **H** The survival curves between high score and low score patients. *p < 0.05, **p < 0.01, ***p< 0.001. SCC: Squamous Cell Carcinoma; AC: Adenocarcinoma; ASC: Adenosquamous Carcinoma

To validate the predictive model's independent prognostic value, we conducted comprehensive bioinformatics analyses. First, clinical sample data from the TCGA cohort were grouped by age, BMI, T stage, N stage, pathological stage, grade, and histological type, and risk scores were computed for each group. The analysis showed that no significant statistical differences were observed in risk scores between groups based on age, BMI, grade, T stage, and histological type (Fig. 5A–D). However, patients with N1 and N2 stages obtained higher risk scores than those with N0 stage (Fig. 5E), and patients with stage III and IV had significantly higher risk scores than those with stage I and II (Fig. 5F). Additionally, no significant differences in risk scores were noted across the groups based on histological type (Fig. 5G). K–M survival curves showed significantly shorter overall survival in high-risk patients than in low-risk patients (Fig. 5H). These results suggest that our predictive model has high prognostic value in advanced pathological stages and LNM status, effectively distinguishing patients with different risk levels and providing critical information for clinical decision-making.

**Fig. 6:**
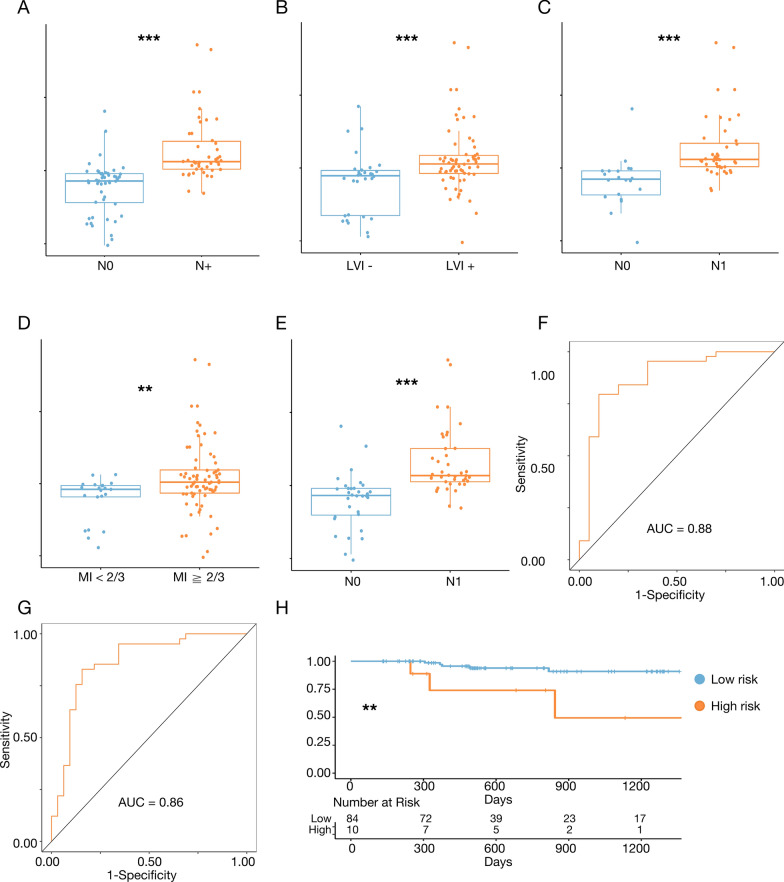
Subgroup analysis in the SYSMH cohort. **A** Riskscore corresponding to each group of N stage (N0 and N +). **B** Riskscore corresponding to each group of lymphovascular invasion status. **C** Riskscore corresponding to each group of lymph status in positive lymphovascular invasion. **D** Riskscore corresponding to each group of myometrial invasion status. **E** Riskscore corresponding to each group of lymph status in deep myometrial invasion. **F** The ROC curves of the prediction model for positive lymphovascular invasion cohort. **G** The ROC curves of the prediction model for deep myometrial invasion cohort. **H** The survival curves between high score and low score patients. *p < 0.05, **p < 0.01, ***p < 0.001. AUC: area under roc curve; LVI: lymphovascular invasion; MI: myometrial invasion

In the external validation cohort from SYSMH, samples were grouped by lymph node status, myometrial invasion, and LVSI. The results showed that lymph node-positive patients obtained higher risk scores than lymph node-negative patients (Fig. 6A). Lymphovascular invasion-positive patients exhibited significantly elevated risk scores compared to those with negative lymphovascular invasion (Fig. 6B), and within the lymphovascular invasion-positive group, lymph node-positive patients had higher risk scores than lymph node-negative patients (Fig. 6C). Patients with deep myometrial invasion obtained higher risk scores than those with superficial invasion (Fig. 6D), and within the deep myometrial invasion group, lymph node-positive patients had higher risk scores than lymph node-negative patients (Fig. 6E). Our model also performed well in distinguishing lymph node status in deep myometrial invasion and lymphovascular invasion-positive patients, with AUC values of 0.86 (95% CI 0.77–0.96) and 0.88 (95% CI 0.77–0.98), respectively (Fig. 6F, G). K–M survival analysis indicated that high-risk patients had a significantly reduced overall survival, consistent with the results from public databases (Fig. 6H).

In summary, these analyses and validation results indicate that our predictive model has high prognostic value in clinical applications for cervical cancer patients, particularly in advanced pathological stages and LNM status. The model not only effectively distinguishes patients with different risk levels but also provides important information for clinical decision-making, guiding individualized treatment strategies.

### Enrichment analysis of DEGs related to LNM in cervical cancer

After DE analysis between high-risk and low-risk groups in the training cohort, we identified 4676 DEGs. To elucidate their associated biological processes and pathways, we performed KEGG and GO enrichment analyses.

**Fig. 7:**
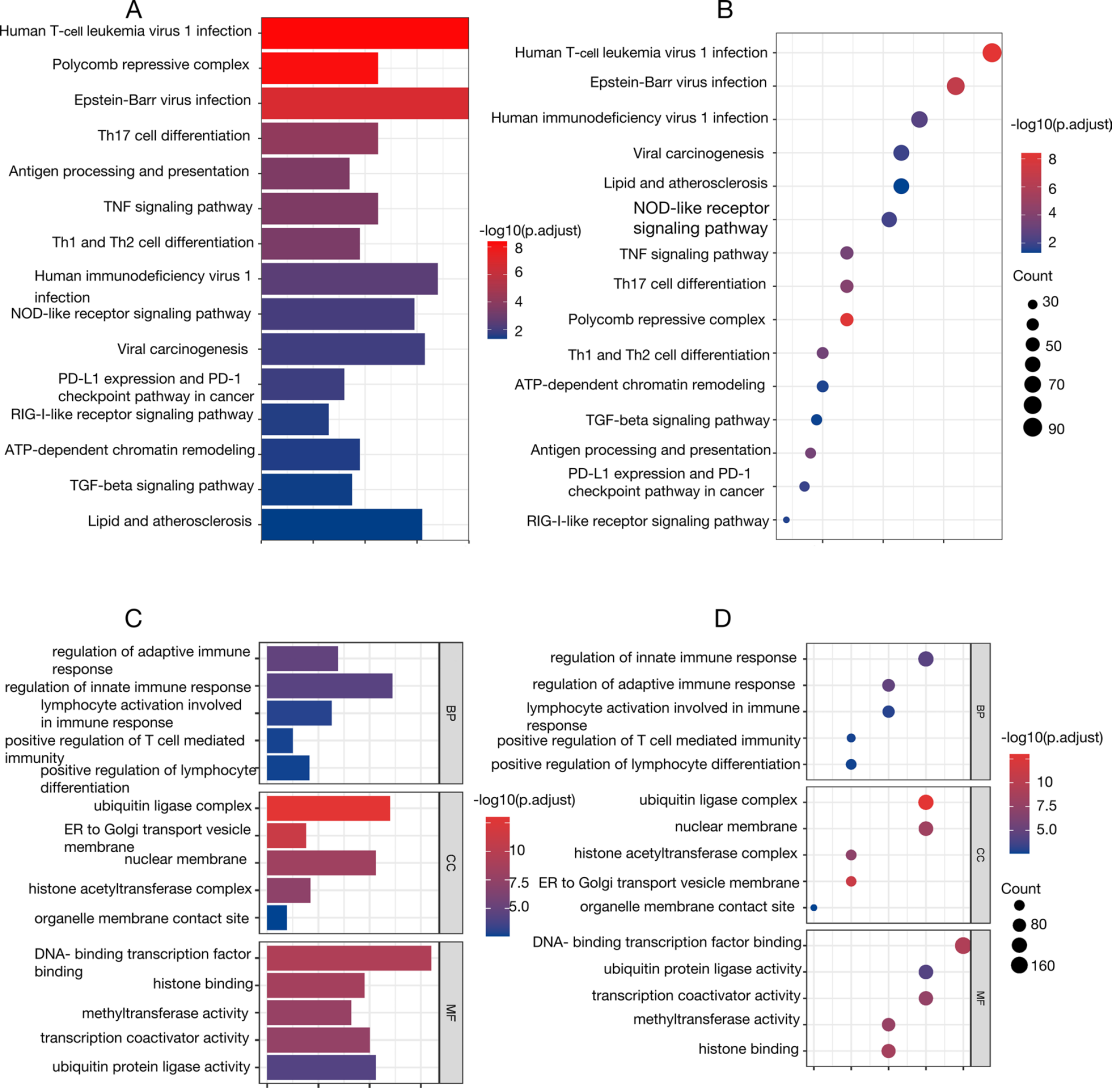
Enrichment analyses of low and high risk group in TCGA cohort

KEGG pathway analysis revealed that these DEGs were mainly engaged in multiple immune-associated biological processes. The significantly enriched pathways included PD-L1 expression, the PD-1 checkpoint pathway in cancer, T cell receptor signaling pathway, natural killer cell-mediated cytotoxicity, cytokine-cytokine receptor interaction, and the differentiation of Th17 cell. These enriched pathways suggest that DEGs related to lymph node metastasis play important roles in immune responses and their regulation (Fig. 7A, B).

GO enrichment analysis further validated these immune-related enrichment results. Significantly enriched processes included type I interferon-mediated signaling pathway, modulation of adaptive immune response, negative regulation of innate immunity, regulation of innate immunity, and enhancement of adaptive immune response. In terms of molecular function, enrichment analysis revealed that these DEGs were mainly associated with antigen binding and T cell receptor binding. Regarding cellular components, significantly enriched regions included MHC class I protein complex and extracellular region (Fig. 7C, D).

Overall, KEGG and GO enrichment analysis results suggest that DEGs related to LNM in cervical cancer are primarily involved in immune responses and immune regulation processes. These results offer significant insights for understanding the biological functions of LNM-related genes in cervical cancer and further validate the applicability and prognostic value of our risk model under different clinical conditions.

## Discussion

Lymph node status significantly influences the prognosis of cervical cancer patients [16]. For patients with early-stage cervical cancer, the recommended surgical approach is radical hysterectomy combined with pelvic lymphadenectomy. However, the NCCN guidelines recommend lymph node biopsy or radiotherapy as the preferred option for locally advanced cancer (IB3, IIA2, IIB, III, IVA) [6]. For women with fertility preservation needs, the presence or absence of LNM directly determines whether fertility-preserving surgery can be performed [17]. Therefore, predicting LNM preoperatively can guide better treatment choices, avoid unnecessary surgeries and related complications, and reduce the financial burden on patients.

Lymph node dissection often leads to complications such as lymphocyst formation, lower extremity edema, vascular injury, nerve injury, and infection [18]. As early-stage cervical cancer has a 15–20% LNM rate, approximately 80% of patients undergoing lymph node dissection may derive little benefit [19, 20]. With advancements in cervical cancer surgery research, more scholars are exploring the value and significance of reducing surgical scope in low-risk cervical cancer patients. For patients with low-risk LNM predicted preoperatively, sentinel lymph node biopsy is a potential alternative to pelvic lymph node dissection. Therefore, pre-treatment lymph node evaluation is key to selecting optimal treatment strategies.

In addition to relying on imaging techniques (such as CT and MRI), many studies have focused on developing models and methods for predicting LNM in cervical cancer. Most of these studies primarily use imaging features and molecular biomarkers to improve predictive accuracy. For example, Liu et al. constructed a LNM prediction model for cervical cancer using MRI radiomic features and machine learning methods, including extreme gradient boosting and support vector machines. These models demonstrated high predictive accuracy and sensitivity in both training and test sets. However, the lack of external validation means that the effectiveness and stability of the models on other datasets remain unverified [21]. Han et al. identified new biomarkers related to LNM in cervical cancer through plasma proteomics analysis. They identified six key biomarkers: SPARC, HPX, VCAM1, TFRC, ERN1, and APMAP. These biomarkers were identified through proteomics analysis and underwent functional annotation and pathway enrichment analysis, providing insights into the mechanisms of LNM. However, the small sample size, which included only 20 patients, limits the representativeness and reliability of the study's findings [22]. Deng et al. integrated various factors such as diagnostic age, histological subtype, tumor grade, tumor size, and FIGO stage using the SEER database and demonstrated high predictive accuracy and sensitivity through internal and external validation [23]. However, the SEER database lacks important data related to LNM, such as LVSI, HPV infection status, and stromal invasion, which may affect the model's predictive accuracy. Furthermore, the SEER database is not a random sample of all cancer patients in the United States, potentially affecting the model's generalizability to other populations [24].

Compared to traditional methods, high-throughput sequencing technology can rapidly and accurately read large amounts of genetic information with high coverage and depth, significantly shortening the research cycle and reducing costs. This technology can be effectively applied to reveal the genetic characteristics of LNM by analyzing gene mutations and regulatory mechanisms in the process of cancer cells spreading from the primary site to lymph nodes. This provides strong data support for understanding the molecular mechanisms of tumor metastasis and developing new therapeutic strategies.

This study systematically analyzed RNA-seq data from 272 cervical cancer patients and developed a predictive model based on gene expression data to assess preoperative LNM risk. The model identified four independent predictive genes (MAPT, EPB41L1, ACSL5, and PRPF4B) and was constructed using LASSO regression and multivariate logistic regression. The results showed that the model performed well in the training cohort, with an AUC of 0.84(95% CI 0.77–0.90). In the external validation cohort, the predictive model's AUC was 0.88(95% CI 0.81–0.96), while CT/MRI's AUC was only 0.71(95% CI 0.62–0.80). The application of the predictive model corrected the misdiagnosis of 81% (22/27) of patients identified by CT/MRI. Specifically, for eight patients with pathologically confirmed negative lymph nodes, imaging suggested LNM, while the predictive model indicated low risk. Of the 19 patients with LNM but negative imaging findings, 14 were identified as high risk by the predictive model. Accuracy increased by 13.9%, sensitivity increased by 21.7%, and specificity increased by 6.3%.

The predictive model developed in this study includes four genes: MAPT, EPB41L1, ACSL5, and PRPF4B. The MAPT gene encodes Tau protein, which is crucial in the process of cancer metastasis. Research indicates that Tau protein is involved in DNA repair and p53 regulation, functions closely related to cancer development and progression [25]. The levels of MAPT expression are linked to specific characteristics of various cancer types, including inflammation, proliferation, and epithelial-mesenchymal transition (EMT). These characteristics are vital for the metastatic spread and invasion of cancer cells. For example, Li et al. reported that elevated MAPT-AS1 expression in breast cancer is associated with enhanced cell proliferation and metastasis. Inhibition of MAPT-AS1 markedly reduced cancer cell proliferation and migration [26]. Research on the EPB41L1 gene's role in cancer cell migration and invasion is currently limited, but preliminary data suggest that changes in EPB41L1 may affect cytoskeletal remodeling, influencing the mobility of cancer cells [27]. The ACSL5 gene encodes an enzyme involved in lipid metabolism, serving a critical function in the metabolic reprogramming of cancer cells. Abnormal lipid metabolism provides energy and structural components for cancer cells, supporting their rapid proliferation and metastasis [28]. And ACSL5 has been found to function as an immune-dependent tumor suppressor. ACSL5 expression enhances tumor sensitivity to PD-1 blockade therapy in vivo and CD8 + T cell-mediated cytotoxicity in vitro by regulating major histocompatibility complex class I (MHC-I)-mediated antigen presentation [29]. PRPF4B plays a critical role in RNA splicing. Aberrant RNA splicing is a hallmark of many cancers, and dysfunction of PRPF4B may promote cancer cell metastasis by altering the expression patterns of key genes. The loss of PRPF4B in breast cancer cell lines Hs578T and MDA-MB-231 significantly inhibited cell migration, almost completely preventing the spread of tumors from the primary site to distant organs, indicating the importance of PRPF4B in metastasis formation [30]. These genes' roles in cancer metastasis provide important directions for future research, and further understanding of their mechanisms may help develop new therapeutic strategies.

We evaluated the independent predictive value of these four genes in both the training and internal validation groups. The results indicated that in the training group, the AUC values for MAPT, PRPF4B, ACSL5, and EPB41L1 in predicting LNM were 0.64 (95% CI 0.53–0.74), 0.7 (95% CI 0.61–0.79), 0.65 (95% CI 0.56–0.75), and 0.66 (95% CI 0.57–0.76), respectively. In the internal validation group, the AUC values for these genes were significantly higher: 0.96 (95% CI 0.93–0.99) for MAPT, 0.93 (95% CI 0.89–0.97) for PRPF4B, 0.96 (95% CI 0.93–0.99) for ACSL5, and 0.93 (95% CI 0.88–0.97) for EPB41L1 (Figure S2).While the AUC values for the individual genes were lower in the training group, we found that the combined predictive model using all four genes demonstrated excellent performance. Although the AUC of the combined predictive model in the internal validation group did not reach the expected high level, this does not imply that the model's predictive capability is weak. In fact, we observed that individual genes demonstrated excellent discriminative ability in the internal validation group. These results further validate the significance of the model and its key genes in predicting LNM in cervical cancer, highlighting the potential and reliability of the model for practical clinical application.

The advantages of this study are: Firstly, the model is based on large-scale gene expression data, and independent predictive genes were selected using rigorous statistical methods, ensuring high accuracy and reliability of the model. Secondly, compared to traditional imaging techniques, the predictive model demonstrated excellent sensitivity and accuracy, helping to reduce clinical misdiagnosis and missed diagnosis. Moreover, by accurately predicting LNM, the model has the potential to reduce overtreatment, optimize therapeutic strategies, and improve patient outcomes. However, there are also some limitations in this study. First, the model was constructed primarily using publicly available databases. While these sources provide valuable insights, they also introduce inherent limitations. The sample size may not be sufficient to fully capture the variability across different populations, leading to potential overfitting. Additionally, publicly available databases often have inherent biases related to data collection methods, population representation, and missing data patterns, which could influence the generalizability of our findings. To ensure the model's broader applicability, validation in larger, multicenter cohorts with diverse demographic and clinical characteristics is essential. Second, although the model performed well in the training cohort and external validation, the AUC value in the internal validation cohort was relatively low, suggesting that further optimization and adjustment of model parameters are needed. Exploration of more potential biomarkers may enhance the model's predictive power and clinical utility.

Additionally, during our research, we found that imaging diagnostics can help correct some misdiagnoses made by the predictive model. Based on this finding, future studies will focus on exploring the integration of imaging data with predictive models. Therefore, future research will focus on developing a multimodal diagnostic system that combines traditional predictive models with imaging techniques. This system will employ cross-validation and complementary mechanisms between deep learning image analysis and model predictions to further improve the preoperative diagnostic accuracy of cervical cancer lymph node metastasis. Furthermore, with the rise of privacy-preserving technologies such as Federated Learning, future research will explore how to share imaging data across multiple centers while protecting patient privacy, thereby improving the generalizability and applicability of diagnostic models [31]. By integrating these cutting-edge technologies, we aim to build an intelligent, precise, and widely adaptable diagnostic system to provide more scientific and personalized decision support for preoperative assessments in cervical cancer.

## Conclusion

In summary, the predictive model developed in this study, based on gene expression data, improves the accuracy and efficiency of preoperative LNM assessments in cervical cancer and has the potential to provide an effective and practical auxiliary tool for clinicians, ultimately improving patient outcomes. Further optimization and validation may lead to the model's widespread clinical application, providing a scientific basis for personalized treatment plans.

## Supplementary Information

Supplementary Material 1

Supplementary Material 2

## Data Availability

Raw RNA-seq data are available from TCGA (https://xenabrowser.net/datapages/) and GEO (https://www.ncbi.nlm.nih.gov/).

## Abbreviations

LNM: Lymph node metastasis
LASSO: The least absolute shrinkage and selection operator
HPV: Human papillomavirus
WHO: World Health Organization
NCCN: National Comprehensive Cancer Network
CT: Computed tomography
MRI: Magnetic resonance imaging
PET: Positron emission tomography
TCGA: The Cancer Genome Atlas
GEO: The Gene Expression Omnibus
TPM: Transcripts per million
PCA: Principal component analysis
DEGs: Differentially expressed genes
DCA: Decision curve analysis
RT-qPCR: Real-time Quantitative Polymerase Chain Reaction
ROC-AUC: The Receiver Operating Characteristic-Area Under the Curve
BMI: Body Mass Index
T: Tumor
N: Node
ANOVA: Analysis of variance
GO: Gene ontology
KEGG: Kyoto encyclopedia of genes and genomes
SD: Standard deviation
IQR: Interquartile range
PPV: Positive predictive value
NPV: Negative predictive value
K-M: Kaplan–Meier
LVSI: Lymphovascular space invasion
EMT: Epithelial-mesenchymal transition
MHC-I: Major histocompatibility complex class I
