# Supplementary material for: A gene-based predictive model for lymph node metastasis in cervical cancer: superior performance over imaging techniques
Source: J Transl Med. 2025 Apr 3;23:397. doi: 10.1186/s12967-025-06327-3 (PMC11969859; doi:10.1186/s12967-025-06327-3)
Supplement: Supplementary file 1 — Supplementary Material 1 [file 12967_2025_6327_MOESM1_ESM.docx]

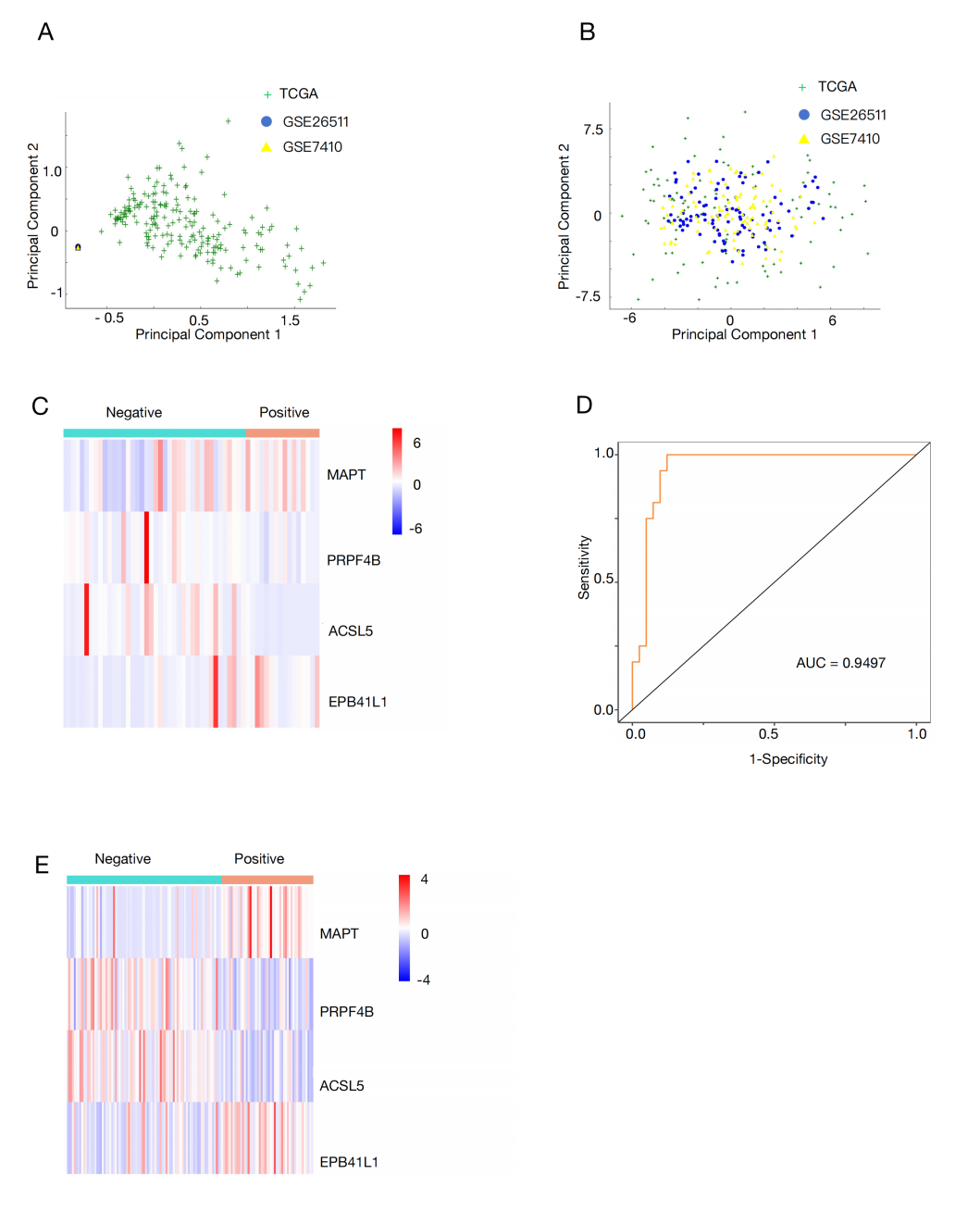


**Supplementary Figure 1. Batch removal and Data validation.**

A: PCA plots describing sample distribution for the 3 cohorts before removing the batch effect. B:PCA plots describing sample distribution for the 3 cohorts after removing the batch effect. C: The RT-qPCR validation of the model feature genes in the tertiary hospital cohort. D: The ROC curves of the prediction model for the tertiary hospital cohort. E: The expression heatmap of four genes in the internal validation cohort.
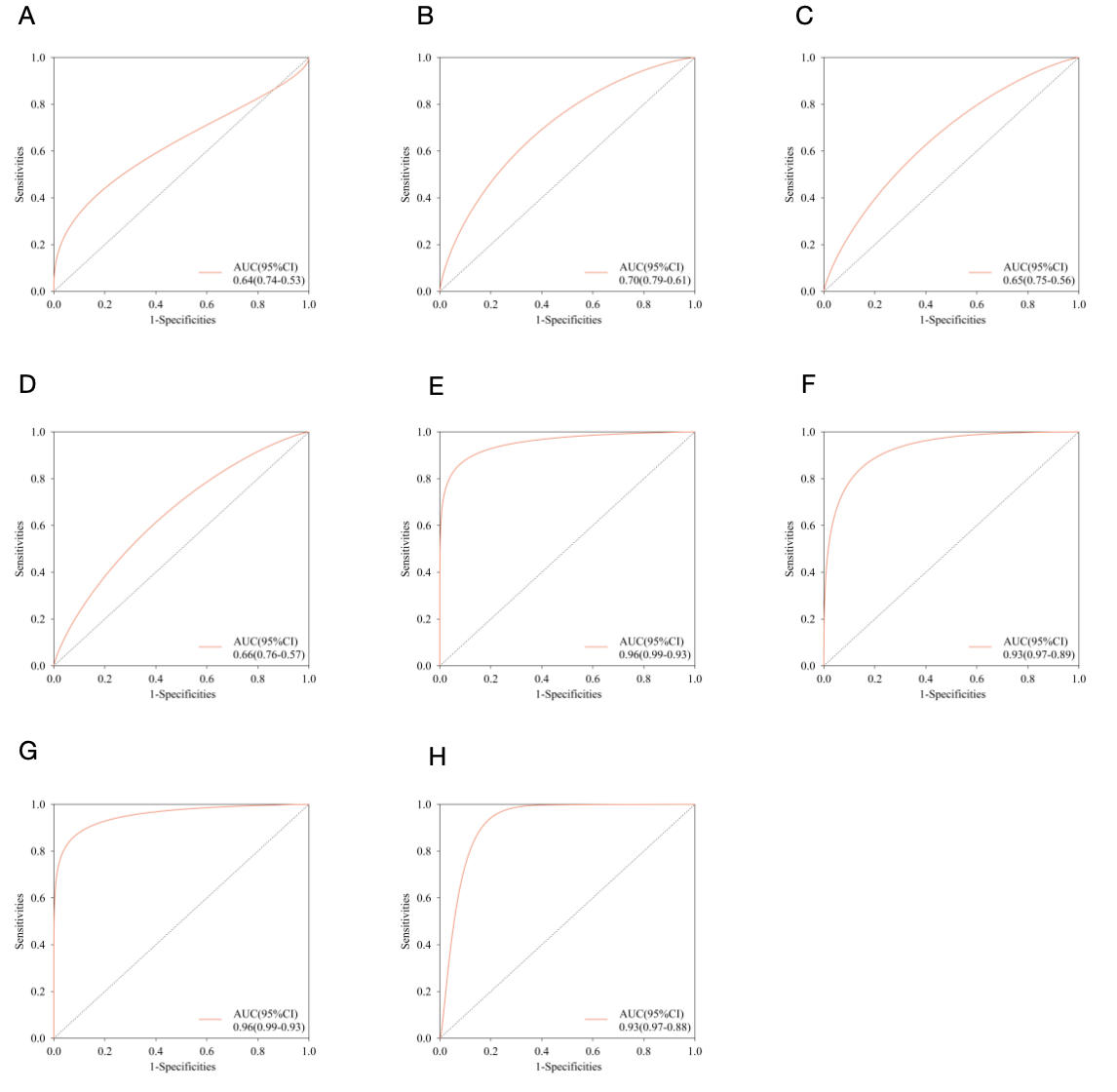


**Supplementary Figure 2.** **ROC curve of genes in risk model for predicting lymph node metastasis.**

A-D: ROC curves of MAPT, PRPF4B, ACSL5, and EPB41L1 in the training cohort. E-H:ROC curves of MAPT, PRPF4B, ACSL5, and EPB41L1 in the internal validation cohort.
