# Supplementary material for: A gene-based predictive model for lymph node metastasis in cervical cancer: superior performance over imaging techniques
Source: J Transl Med. 2025 Apr 3;23:397. doi: 10.1186/s12967-025-06327-3 (PMC11969859; doi:10.1186/s12967-025-06327-3)
Supplement: Supplementary file 2 — Supplementary Material 2 [file 12967_2025_6327_MOESM2_ESM.docx]

**Table S1** Primer sequences (5′-3′) for qRT-PCR.

| MAPT | GGTGGCCAGGTGGAAGTAAA | CCAGGGACCCAATCTTCGAC |
| --- | --- | --- |
| PRPF4B | CCCATTAGAAGGAGGTCTCGTTC | TTCGCAAGCGTGATCTGCTCCT |
| ACSL5 | GGACTCGCTGTGTCTGACAA | AGGTCTTCTGGGCTAGGAGG |
| EPB41L1 | AGGAAACCACGCCGAGACACAA | GGTGGATGAGTTTGCTGTTGGG |

**Table S2** Differentially expressed genes between LN-positive patients and LN-negative patients.

| Symbol | logFC | AveExpr | t | P.Value | adj.P.Val | B |
| --- | --- | --- | --- | --- | --- | --- |
| CLIC3 | 0.562772557 | -0.00686127 | 3.693884561 | 0.00029191 | 0.957712014 | -3.160519763 |
| MAPT | 0.568979976 | -0.016638315 | 3.61281623 | 0.00039173 | 0.957712014 | -3.224843374 |
| PRPF4B | -0.621012245 | -0.110870743 | -3.557365185 | 0.000477671 | 0.957712014 | -3.268212964 |
| ANKRD28 | -0.552942253 | -0.164592291 | -3.535738155 | 0.000515767 | 0.957712014 | -3.284988039 |
| TRIM8 | -0.61799097 | 0.013550741 | -3.496656648 | 0.00059195 | 0.957712014 | -3.315100838 |
| ACSL5 | -0.601994589 | -0.016286775 | -3.304498649 | 0.001145839 | 0.957712014 | -3.459329089 |
| IVL | 0.542407247 | -0.024138738 | 3.301810891 | 0.001156242 | 0.957712014 | -3.461300528 |
| EPHA1 | 0.540303338 | -0.032202497 | 3.288300685 | 0.001209877 | 0.957712014 | -3.471190648 |
| DZIP1L | -0.595684165 | 0.034965354 | -3.257145985 | 0.001342515 | 0.957712014 | -3.493873229 |
| THOC2 | -0.546461413 | -0.108275923 | -3.253389096 | 0.001359394 | 0.957712014 | -3.49659674 |
| LETMD1 | -0.597247943 | -0.003649048 | -3.242935659 | 0.001407401 | 0.957712014 | -3.504161493 |
| DCXR | 0.548365814 | 0.033875889 | 3.111168188 | 0.002163971 | 0.957712014 | -3.597817661 |
| MINPP1 | -0.546661307 | -0.098700465 | -3.106378147 | 0.002197518 | 0.957712014 | -3.601162401 |
| CLDN9 | 0.572324154 | 0.027769014 | 3.0857105 | 0.00234785 | 0.957712014 | -3.615545348 |
| GALK1 | 0.534355354 | 0.087701313 | 3.063852472 | 0.002517128 | 0.957712014 | -3.630670505 |
| PHC2 | -0.51418627 | -0.097550497 | -3.059916052 | 0.002548784 | 0.957712014 | -3.633384958 |
| TMEM17 | -0.525950802 | -0.101287593 | -3.057827311 | 0.00256573 | 0.957712014 | -3.634824126 |
| PIGC | -0.507285177 | -0.043814665 | -2.972780272 | 0.003350627 | 0.957712014 | -3.692728309 |
| LMX1B | 0.537866731 | -0.003098255 | 2.963946146 | 0.003443695 | 0.957712014 | -3.698664855 |
| EPB41L1 | 0.503685246 | 0.00325246 | 2.916747059 | 0.003982384 | 0.957712014 | -3.730131342 |
| WDR19 | -0.542414364 | -0.046931988 | -2.897612836 | 0.004221948 | 0.957712014 | -3.742766488 |
| ECD | -0.506126471 | -0.007732591 | -2.87197636 | 0.004563585 | 0.957712014 | -3.759585174 |
| SLC39A14 | -0.512297106 | -0.051763712 | -2.867678006 | 0.004623277 | 0.957712014 | -3.762392702 |
| SQLE | 0.507739946 | -0.065680752 | 2.852246 | 0.004843506 | 0.957712014 | -3.772442936 |
| TRAPPC2 | -0.509767725 | -0.006059936 | -2.832650888 | 0.005136926 | 0.957712014 | -3.785138032 |
| NHLRC2 | -0.511568096 | 0.001643067 | -2.704029794 | 0.007500115 | 0.957712014 | -3.866606412 |

**Table S3** Multivariate logistic regression of 18 genes from LASSO results

| Symbol | Pr(>\|z\|) | OR | LCI | UCI |
| --- | --- | --- | --- | --- |
| CLIC3 | 0.155519606 | 1.773252157 | 0.822479149 | 4.063560058 |
| MAPT | 0.014034513 | 3.243403248 | 1.373957127 | 9.105367515 |
| PRPF4B | 0.038679648 | 0.324889661 | 0.104435864 | 0.910051591 |
| ANKRD28 | 0.594281461 | 0.759954648 | 0.268300454 | 2.085380435 |
| ACSL5 | 0.026936825 | 0.394145632 | 0.159463971 | 0.829086274 |
| IVL | 0.599111666 | 1.212697124 | 0.616592291 | 2.638133309 |
| EPHA1 | 0.083101082 | 1.928209563 | 0.938897959 | 4.220543068 |
| DZIP1L | 0.507186739 | 0.788682415 | 0.361167332 | 1.487526132 |
| THOC2 | 0.900043735 | 0.929187412 | 0.279105497 | 2.870808769 |
| DCXR | 0.080954275 | 1.63403354 | 0.938838306 | 2.885896245 |
| MINPP1 | 0.0653449 | 2.12536831 | 0.966836794 | 4.938706328 |
| PHC2 | 0.778893968 | 1.099163219 | 0.556655868 | 2.129245365 |
| PIGC | 0.744286705 | 1.13898611 | 0.508270908 | 2.495212411 |
| EPB41L1 | 0.026181583 | 2.136139339 | 1.114080157 | 4.311692729 |
| WDR19 | 0.745036474 | 0.887119908 | 0.42436277 | 1.849081731 |
| SLC39A14 | 0.125703197 | 0.564492757 | 0.25292456 | 1.130176719 |
| TRAPPC2 | 0.182034356 | 0.562930769 | 0.229224984 | 1.265366473 |
| NHLRC2 | 0.874594201 | 0.929553684 | 0.376400641 | 2.339064406 |

**Table S4** logistic regression in SYSMH cohort

| Variables | Univariate logistic regression | | | | | Multivariate logistic regression | | | | | | | | | |
| --- | --- | --- | --- | --- | --- | --- | --- | --- | --- | --- | --- | --- | --- | --- | --- |
|  | β | S.E | Z | *P* | OR (95%CI) |  | β | S.E | Z | *P* | OR (95%CI) |  |  |  |  |
| Age | -0.01 | 0.02 | -0.61 | 0.542 | 0.99 (0.95 ~ 1.03) |  |  |  |  |  |  |  |  |  |  |
| BMI | -0.01 | 0.07 | -0.15 | 0.881 | 0.99 (0.87 ~ 1.13) |  |  |  |  |  |  |  |  |  |  |
| Risk score | 0.01 | 0.00 | 3.84 | **<.001** | 1.01 (1.01 ~ 1.01) |  | 0.01 | 0.00 | 3.39 | **<.001** | 1.01 (1.01 ~ 1.01) |  |  |  |  |
| Myometrial-Invasion |  |  |  |  |  |  |  |  |  |  |  |  |  |  |  |
| <2/3 |  |  |  |  | 1.00 (Reference) |  |  |  |  |  | 1.00 (Reference) |  |  |  |  |
| ≥2/3 | 1.41 | 0.56 | 2.50 | **0.012** | 4.10 (1.36 ~ 12.38) |  | 0.55 | 0.91 | 0.60 | 0.548 | 1.73 (0.29 ~ 10.31) |  |  |  |  |
| N |  |  |  |  |  |  |  |  |  |  |  |  |  |  |  |
| N0 |  |  |  |  | 1.00 (Reference) |  |  |  |  |  |  |  |  |  |  |
| N1+2 | 53.13 | 73479.32 | 0.00 | 0.999 | 118848048318589161177088.00 (0.00 ~ Inf) |  |  |  |  |  |  |  |  |  |  |
| Type |  |  |  |  |  |  |  |  |  |  |  |  |  |  |  |
| AC |  |  |  |  | 1.00 (Reference) |  |  |  |  |  |  |  |  |  |  |
| ASC | 0.41 | 0.84 | 0.48 | 0.630 | 1.50 (0.29 ~ 7.81) |  |  |  |  |  |  |  |  |  |  |
| SCC | 0.47 | 0.52 | 0.90 | 0.369 | 1.59 (0.58 ~ 4.40) |  |  |  |  |  |  |  |  |  |  |
| Grade |  |  |  |  |  |  |  |  |  |  |  |  |  |  |  |
| G1 |  |  |  |  | 1.00 (Reference) |  |  |  |  |  |  |  |  |  |  |
| G2 | 16.50 | 1199.77 | 0.01 | 0.989 | 14607936.85 (0.00 ~ Inf) |  |  |  |  |  |  |  |  |  |  |
| G3 | 16.66 | 1199.77 | 0.01 | 0.989 | 17270467.22 (0.00 ~ Inf) |  |  |  |  |  |  |  |  |  |  |
| LVI |  |  |  |  |  |  |  |  |  |  |  |  |  |  |  |
| 0 |  |  |  |  | 1.00 (Reference) |  |  |  |  |  | 1.00 (Reference) |  |  |  |  |
| 1 | 3.43 | 0.78 | 4.39 | **<.001** | 30.80 (6.68 ~ 142.07) |  | 4.05 | 1.29 | 3.13 | **0.002** | 57.59 (4.57 ~ 726.07) |  |  |  |  |
| M |  |  |  |  |  |  |  |  |  |  |  |  |  |  |  |
| 0 |  |  |  |  | 1.00 (Reference) |  |  |  |  |  |  |  |  |  |  |
| 1 | 16.70 | 1199.77 | 0.01 | 0.989 | 17887269.48 (0.00 ~ Inf) |  |  |  |  |  |  |  |  |  |  |
| CT/MRI |  |  |  |  |  |  |  |  |  |  |  |  |  |  |  |
| 0 |  |  |  |  | 1.00 (Reference) |  |  |  |  |  | 1.00 (Reference) |  |  |  |  |
| 1 | 1.96 | 0.49 | 4.01 | **<.001** | 7.11 (2.72 ~ 18.55) |  | 1.38 | 0.73 | 1.91 | 0.056 | 3.99 (0.96 ~ 16.53) |  |  |  |  |
| T |  |  |  |  |  |  |  |  |  |  |  |  |  |  |  |
| 1b1 |  |  |  |  | 1.00 (Reference) |  |  |  |  |  |  |  |  |  |  |
| 1b2 | 15.82 | 1385.38 | 0.01 | 0.991 | 7413802.47 (0.00 ~ Inf) |  |  |  |  |  |  |  |  |  |  |
| 1b3 | 16.23 | 1385.38 | 0.01 | 0.991 | 11179543.40 (0.00 ~ Inf) |  |  |  |  |  |  |  |  |  |  |
| 2a1 | 16.57 | 1385.38 | 0.01 | 0.990 | 15651360.76 (0.00 ~ Inf) |  |  |  |  |  |  |  |  |  |  |
| 2a2 | 17.66 | 1385.38 | 0.01 | 0.990 | 46954082.28 (0.00 ~ Inf) |  |  |  |  |  |  |  |  |  |  |
| 2b | 17.04 | 1385.38 | 0.01 | 0.990 | 25042177.22 (0.00 ~ Inf) |  |  |  |  |  |  |  |  |  |  |
| Stage |  |  |  |  |  |  |  |  |  |  |  |  |  |  |  |
| IB1 |  |  |  |  | 1.00 (Reference) |  |  |  |  |  |  |  |  |  |  |
| IB2 | 19.62 | 27826.06 | 0.00 | 0.999 | 332326854.51 (0.00 ~ Inf) |  |  |  |  |  |  |  |  |  |  |
| IB3 | 0.00 | 30662.82 | 0.00 | 1.000 | 1.00 (0.00 ~ Inf) |  |  |  |  |  |  |  |  |  |  |
| IIA1 | -0.00 | 55652.11 | -0.00 | 1.000 | 1.00 (0.00 ~ Inf) |  |  |  |  |  |  |  |  |  |  |
| IIA2 | -0.00 | 34079.82 | -0.00 | 1.000 | 1.00 (0.00 ~ Inf) |  |  |  |  |  |  |  |  |  |  |
| IIB | 0.00 | 35197.49 | 0.00 | 1.000 | 1.00 (0.00 ~ Inf) |  |  |  |  |  |  |  |  |  |  |
| IIIC1 | 45.13 | 29027.73 | 0.00 | 0.999 | 39869252193334804480.00 (0.00 ~ Inf) |  |  |  |  |  |  |  |  |  |  |
| IIIC2 | 45.13 | 33258.50 | 0.00 | 0.999 | 39869252211453345792.00 (0.00 ~ Inf) |  |  |  |  |  |  |  |  |  |  |
| IV | 45.13 | 36810.41 | 0.00 | 0.999 | 39869255178664034304.00 (0.00 ~ Inf) |  |  |  |  |  |  |  |  |  |  |

Abbreviation:

β：Beta coefficient, S.E:Standard Error, Z:Z-score, P:P-value, OR (95%CI):Odds Ratio (95% Confidence Interval), BMI:Body Mass Index, N:Node, AC:Adenocarcinoma, ASC: Adenosquamous carcinoma, SCC: Squamous cell carcinoma, LVI:Lymphovascular Invasion, M:Metastasis, CT:Computed Tomography, MRI:Magnetic resonance imaging, T:Tumor.

**Table S5** Clinical Information Summary of the SYSMH Cohort.

| Variables | Total (n = 94) |
| --- | --- |
|  |  |
| Age, Mean ± SD | 51.68 ± 10.64 |
| BMI, Mean ± SD | 23.49 ± 3.10 |
| Myometrial-Invasion, n(%) |  |
| <2/3 | 21 (22.34) |
| ≥2/3 | 73 (77.66) |
| Type, n(%) |  |
| AC | 20 (21.28) |
| ASC | 8 (8.51) |
| SCC | 66 (70.21) |
| Grade, n(%) |  |
| G1 | 4 (4.26) |
| G2 | 29 (30.85) |
| G3 | 61 (64.89) |
| Lymphovascular Invasion, n(%) |  |
| Negative | 30 (31.91) |
| Positive | 64 (68.09) |
| T, n(%) |  |
| T1 | 55 (58.51) |
| T2 | 39 (41.49) |
| N, n(%) |  |
| Negative | 48 (51.06) |
| Positive | 46 (48.94) |
| M, n(%) |  |
| Negative | 90 (95.74) |
| Positive | 4 (4.26) |
| Stage, n(%) |  |
| I | 37(39.36) |
| II | 12 (12.77) |
| III | 41 (43.62) |
| IV | 4 (4.26) |

Abbreviation:

SD:Standard Deviation,AC:Adenocarcinoma，ASC：Adenosquamous carcinoma，SCC：Squamous cell carcinoma，N:Node,T:Tumor,M:Metastasis

**Table S6** Clinical Information Summary of the TCGA Cohort.

| Variables | Total (n = 193) |
| --- | --- |
|  |  |
| Height, Mean ± SD | 161.63 ± 7.60 |
| Weight, Mean ± SD | 73.62 ± 22.73 |
| Age, Mean ± SD | 46.40 ± 12.98 |
| N, n(%) |  |
| N0 | 133 (68.91) |
| N1 | 60 (31.09) |
| T, n(%) |  |
| T1 | 132 (68.39) |
| T2 | 51 (26.42) |
| T3 | 8 (4.15) |
| T4 | 2 (1.04) |
| M, n(%) |  |
| M0 | 189 (97.93) |
| M1 | 4 (2.07) |
| Stage, n(%) |  |
| I | 129 (66.84) |
| II | 37 (19.17) |
| III | 23 (11.92) |
| IV | 4 (2.07) |
| Grade, n(%) |  |
| G1 | 14 (7.25) |
| G2 | 86 (44.56) |
| G3 | 83 (43.01) |
| G4 | 1 (0.52) |
| GX | 9 (4.66) |

Abbreviation:

SD:Standard Deviation,N:Node,T:Tumor,M:Metastasis

**Table S7** Clinical Information Summary of the GSE7410 Cohort

| Variables | Total (n = 40) |
| --- | --- |
|  |  |
| Figo stage, n(%) |  |
| IB1 | 19 (47.50) |
| IB2 | 7 (17.50) |
| IIA | 14 (35.00) |
| Lymphnode, n(%) |  |
| Negative | 21 (52.50) |
| Positive | 19 (47.50) |

**Table S8** Clinical Information Summary of the GSE26511 Cohort

| Variables | Total (n = 39) |
| --- | --- |
|  |  |
| Age, Mean ± SD | 58.12 ± 76.29 |
| Lymph Node, n(%) |  |
| Negative | 20 (51.28) |
| Positive | 19 (48.72) |
| FIGO stage, n(%) |  |
| 1b1 | 21 (53.85) |
| 1b2 | 11 (28.21) |
| 2a | 7 (17.95) |

Abbreviation:

SD:Standard Deviation
